# Differentiating Medicated Patients Suffering from Major Depressive Disorder from Healthy Controls by Spot Urine Measurement of Monoamines and Steroid Hormones

**DOI:** 10.3390/ijerph15050865

**Published:** 2018-04-26

**Authors:** Chandra S. Wijaya, Jovia J. Z. Lee, Syeda F. Husain, Cyrus S. H. Ho, Roger S. McIntyre, Wilson W. Tam, Roger C. M. Ho

**Affiliations:** 1 Singapore Technology Center, Panasonic Industrial Devices Singapore, Singapore 469269, Singapore; chandra.wijaya@sg.panasonic.com (C.S.W.); jovia.leejz@sg.panasonic.com (J.J.Z.L.); 2 Department of Psychological Medicine, Yong Loo Lin School of Medicine, National University of Singapore, Singapore 119228, Singapore; e0157451@u.nus.edu (S.F.H.); su_hui_ho@nuhs.edu.sg (C.S.H.H.); 3 Department of Psychological Medicine, National University Health System, Singapore 119228, Singapore; 4 Institute of Medical Science, University of Toronto, Toronto, ON M5S 1A8, Canada; Roger.McIntyre@uhn.ca; 5 Mood Disorders Psychopharmacology Unit, University Health Network, Toronto, ON M5G 2C4, Canada; 6 Department of Psychiatry, University of Toronto, Toronto, ON M5T 1R8, Canada; 7 Department of Toxicology and Pharmacology, University of Toronto, Toronto, ON M5S 1A8, Canada; 8 Alice Lee School of Nursing, Yong Loo Lin School of Medicine, National University of Singapore, Singapore 117597, Singapore; nurtwsw@nus.edu.sg

**Keywords:** major depressive disorder, monoamine, steroid hormone, spot urine

## Abstract

*Introduction*: Major Depressive Disorder (MDD) is a common psychiatric disorder. Currently, there is no objective, cost-effective and non-invasive method to measure biological markers related to the pathogenesis of MDD. Previous studies primarily focused on urinary metabolite markers which are not proximal to the pathogenesis of MDD. Herein, we compare urinary monoamines, steroid hormones and the derived ratios amongst MDD when compared to healthy controls. *Methods*: Morning urine samples of medicated patients suffering from MDD (*n* = 47) and healthy controls (*n* = 41) were collected. Enzyme-linked immunosorbent assay (ELISA) was performed to measure five biomarkers: cortisol, dopamine, noradrenaline, serotonin and sulphate derivative of dehydroepiandrosterone (DHEAS). The mean urinary levels and derived ratios of monoamines and steroid hormones were compared between patients and controls to identify potential biomarkers. The receiver operative characteristic curve (ROC) analysis was conducted to evaluate the diagnostic performance of potential biomarkers. *Results*: Medicated patients with MDD showed significantly higher spot urine ratio of DHEAS/serotonin (1.56 vs. 1.19, *p* = 0.004) and lower ratio of serotonin/dopamine (599.71 vs. 888.60, *p* = 0.008) than healthy controls. A spot urine serotonin/dopamine ratio cut-off of >667.38 had a sensitivity of 73.2% and specificity of 51.1%. *Conclusions*: Our results suggest that spot urine serotonin/dopamine ratio can be used as an objective diagnostic method for adults with MDD.

## 1. Introduction

Major Depressive Disorder (MDD) is a common psychiatric disorder. The point prevalence is 1.8–3.2% for men and 2.0–9.3% for women [1]. Besides low mood, MDD is associated with negative cognitive function [2], maladaptive thoughts [3] and poor quality of life [4]. The mean annual total costs of managing MDD per patient was estimated to be US$7638 [5]. The response rate to antidepressant treatment is around 65% [6]. Despite the advances in antidepressant treatment, there is a lack of cost-effective and non-invasive method to obtain biomarkers to differentiate medicated patients with MDD from unaffected individuals [7,8].

Collection of urine is a non-invasive method and readily accepted. Previous studies have identified urinary metabolite abnormalities in patients suffering from MDD. Zheng et al. (2013) [9] identified five urinary metabolite biomarkers (i.e., alanine, formate, malonate, m-hydroxyphenylacetate, and *N*-methynicotinamide) that differentiated depressed participants and healthy controls. In a sample of individuals with postpartum depression, a panel of five potential biomarkers—1-methylhistidine, α-glucose, dimethylamine, formate, and succinate—were identified [10]. Moreover, in a sample of individuals with post-stroke depression, a panel of six metabolites—azelaic acid, glyceric acid, pseudouridine, 5-hydrocyhexanoic acid, tyrosine and phenylalanine—were identified [11]. For elderly, mono-(3-carboxypropyl) phthalate, mono(carboxypropyl) phthalate and mono-*n*-butyl phthalate were positively associated with the risk of depression in elderly population [12].

Limitations of the foregoing studies is that some biomarkers did not show significant difference between patients with MDD and healthy controls in univariate analysis but were included in the panel. The significant findings with respect to combinatorial metabolites were delimited to multivariate analysis [9,11]. Second, some biomarkers are energy metabolites related to the tricarboxylic acid cycle and glucose metabolism. Psychiatric disorders are associated with increased incidence of metabolic syndrome [13]. Consequently, other confounding factors including alcohol misuse, binge eating, sedentary lifestyle, and hormonal imbalances [14], which could affect MDD and energy metabolites, contribute to a spurious association between MDD and energy metabolites. Similarly, phthalate exposure is associated with oxidative stress [11] and inflammation [15], leading to adverse health outcomes including asthma [16,17,18], pulmonary diseases [19,20] and obesity [21,22] but not specifically to MDD The excretion oxidative stress biomarker, F_2_ isoprostane was found to be inversely correlated with reduction in severity of depression [23]. The association between oxidative stress markers and MDD could be confounded by inflammation [15]. Most importantly, these biomarkers do not play a role in the pathogenesis of MDD and the panel of metabolites varied significantly from study to study. From the cost perspective, the above urinary metabolite biomarkers were analysed by liquid chromatography mass spectroscopy [24] which is not available in most clinical laboratories. This limits the clinical application of above urinary metabolites and oxidative stress biomarkers and translation of such research findings.

The monoamine hypothesis posits that MDD is associated with relative depletion of monoamines including catecholamines (e.g., dopamine and noradrenaline) and tryptamine (e.g., serotonin). In addition, disturbances of the hypothalamic–pituitary–adrenal axis was reported in MDD with high level of cortisol secretion are observed in MDD [1]. In addition to cortisol, the adrenal glands also produce the steroid hormone, dehydroepiandrosterone (DHEA) and its sulphate derivative DHEAS [25]. The production of DHEAS is a compensatory mechanism to counteract the negative effect of MDD. Previous research found that antidepressant treatment and psychotherapy could alter urinary serotonin levels [26,27]. In developed countries with accessible mental health services, patients suffering from MDD often receive antidepressant treatment. It remains unknown whether medicated patients with MDD would have different urinary levels of monoamines and steroid hormones as compared to healthy controls. The application of derived ratio such as sodium/potassium (Na/K) ratio in spot urine could predict diuretic response [28] and it is worthwhile to compare derived ratios based on monoamines and steroid hormones in medicated patients with MDD and healthy controls.

The aim of our study herein was to assess readily available spot urine levels of monoamines and steroid hormones. Towards this aim, we compared the spot urine levels of monoamines and steroid hormones between medicated patients with MDD and healthy controls. The present study was designed to evaluate whether derived ratios such as DHEAS/serotonin, DHEAS/dopamine and serotonin/dopamine could differentiate medicated patients with MDD and healthy controls.

## 2. Materials and Methods

### 2.1. Patients and Healthy Controls

This cross-sectional study included medicated patients with MDD and healthy controls. All patients received antidepressant treatment at National University Hospital (Singapore) between August 2016 and March 2017. Two experienced psychiatrists (R.C.H. and C.S.H.) were in charge recruiting patients diagnosed with MDD. The two psychiatrists provided information of duration of depressive disorder and type of antidepressant treatment. All healthy controls were recruited from the community. For inclusion, both patients and healthy controls must be aged 21 years older. They must have the capacity to sign a written informed consent and agree to participate in this study. For patients, they must have fulfilled the DSM-IV or DSM-5 criteria of MDD during the course of depressive illness. The patients must receive antidepressant treatment during the time of study.

For exclusion criteria, both patients and healthy controls should not be diagnosed with psychiatric disorders including schizophrenia, schizoaffective disorder, bipolar disorder, intellectual disability, substance abuse and dementia, as well as chronic medical disorders including endocrine disorders, cardiovascular disorders, diseases related to central nervous system and rheumatological disorders. The patients and healthy controls could not receive psychotherapy at the time of participation in the study. The patients should not receive electroconvulsive therapy at the time of participation.

Demographic data on the participants were collected using a questionnaire that included questions on age, gender and ethnicity. The 21-item Depression, Anxiety and Stress Scale (DASS) was used to measure severity of depression, anxiety and stress levels. Items are rated on a 4-point scale using a time-frame of “over the past week”. Each of the three DASS-21 scales contains 7 items, divided into subscales with similar content. The depression scale assesses dysphoria, hopelessness, devaluation of life, self-deprecation, lack of interest/involvement, anhedonia and inertia. The anxiety scale assesses autonomic arousal, skeletal muscle effects, situational anxiety, and subjective experience of anxious affect. The stress scale is sensitive to levels of chronic nonspecific arousal. The 21-item of DASS questionnaire was validated in the Singaporean population and the internal consistency of the scale was excellent for Singaporean population (*α* = 0.95) [2]. 

This study protocol conformed to the ethical guidelines of the Declaration of Helsinki and was approved by the institutional ethics committee (The Domain Specific Review Board). Written informed consent was obtained from each participant.

### 2.2. Measurement of Monoamines and Steroid Hormones in Spot Urine

Fasting urine samples of participants were collected between 8:00 a.m. to 11:00 a.m. in a 60 mL urine container. Collected urine samples were stored in an icebox during transportation and then stored in a refrigerator with temperature set at 4 °C before processing on the same day. Each collected urine sample was then centrifuged at 6000 RPM for 9 min and its supernatant was decanted into five separated 1.5 mL microcentrifuge tubes for the testing of the 5 biomarkers (i.e., cortisol, dopamine, noradrenaline, serotonin and DHEAS). 

Five ELISA kits (Kono Biotech Co., Ltd., Jiaxing, China) were used to quantify the levels of cortisol, dopamine, noradrenaline, serotonin and DHEAS in the urine. The dilution factors used for cortisol, dopamine, noradrenaline, serotonin and DHEAS were 4, 4, 1, 4 and 4. respectively. The dilution factor for noradrenaline was 1 because the concentration of the noradrenaline was too low. For biomarkers with dilution factor of 4, 250 µL of urine samples were added into 750 µL of sample diluent. For biomarker with dilution factor of 1, 250 µL of urine samples were added into 250 µL of sample diluent. 

### 2.3. Statistical Analysis

The demographics, DASS scores, levels of monoamines and steroid hormones as well as the derived ratios were compared between medicated patients with MDD and healthy controls. For continuous variables, *t*-test and Mann–Whitney U test were used for parametric and non-parametric data, respectively. For categorical variables, chi square test was used. The receiver-operating characteristic (ROC) curve analysis was conducted to evaluate the diagnostic performance of the urinary markers and derived ratios in diagnosing patients with MDD. To assess the diagnostic performance of these markers, the ROC curve analysis was conducted to calculate the area under the curve (AUC), sensitivity and specificity.

## 3. Results

### 3.1. Baseline Characteristics of Patients and Healthy Controls

Table 1 compares the demographics and clinical backgrounds of medicated patients suffering from MDD (*n* = 47) and healthy controls (*n* = 41). The mean patient age and control age were 40.65 and 44.6 years, respectively, and there was no significant difference (*p* = 0.104). The two groups were demographically matched in gender (*p* = 0.279) and ethnicity (*p* = 0.453). The medicated subjects with depression demonstrated significantly higher depression score (19.29 vs. 2.29, *p* < 0.001), anxiety score (18.86 vs. 2.93, *p* < 0.001) and stress score (21.26 vs. 5.29, *p* < 0.001) as compared to healthy controls. In terms of severity, 59.5% of medicated patients suffered from moderate to extremely severe depression, 78.8% suffered from moderate to extremely severe anxiety and 55.3% suffered from moderate to extremely severe stress. The proportion was significantly higher than healthy controls (*p* < 0.001). None of the healthy controls suffered from moderate to extremely severe depression, anxiety and stress [29]. The mean duration of depression (±standard deviation) of medicated patients was 48.83 months ±54.54.

Thirty-four (72.3%) medicated patients were treated with one antidepressant only. Thirteen (27.7%) medicated patients were treated with two antidepressants. Fourteen (29.8%) medicated patients were treated with both antidepressant and antipsychotic drugs. Of the patients treated with selective serotonin reuptake inhibitors (SSRIs), 7 (14.9%) patients received fluoxetine (M = 35.71 mg/day, SD = 22.99), 12 (25.5%) patients received fluvoxamine (M = 106.25 mg/day, SD = 69.19), 5 (10.6%) patients received escitalporam (M = 16 mg/day, SD = 5.48), 4 (8.5%) patients received paroxetine (M = 25 mg/day, SD = 17.6) and 4 (8.5%) patients received sertraline (M = 56.25 mg/day, SD = 31.46). Of the patients treated with serotonin and noradrenaline reuptake inhibitor (SNRI), two (4.3%) patients received venlafaxine (M = 187.5 mg/day, SD = 53.03). Of the patients treated with noradrenergic and specific serotoninergic antidepressant (NaSSA), 17 (36.2%) patients received mirtazapine (M = 18.97 mg/day, SD = 11.29). Of the patients treated with dopamine and noradrenaline reuptake inhibitor (DNRI), three (6.4%) patients received bupropion (150 mg/day). Of the patients treated with new antidepressants, three (6.4%) patients received agomelatine (M = 41.67 mg/day, SD = 14.43) and one (2.1%) patient received vortioxetine (10 mg/day). Of the patients treated with antipsychotic drug, 13 (27.7%) patients received quetiapine (M = 146.15 mg/day, SD = 110.21). Table S1 provides more information about the antidepressant treatment.

### 3.2. Levels of Catecholamine and Steroid Hormone in Spot Urine

**Figure 1:**
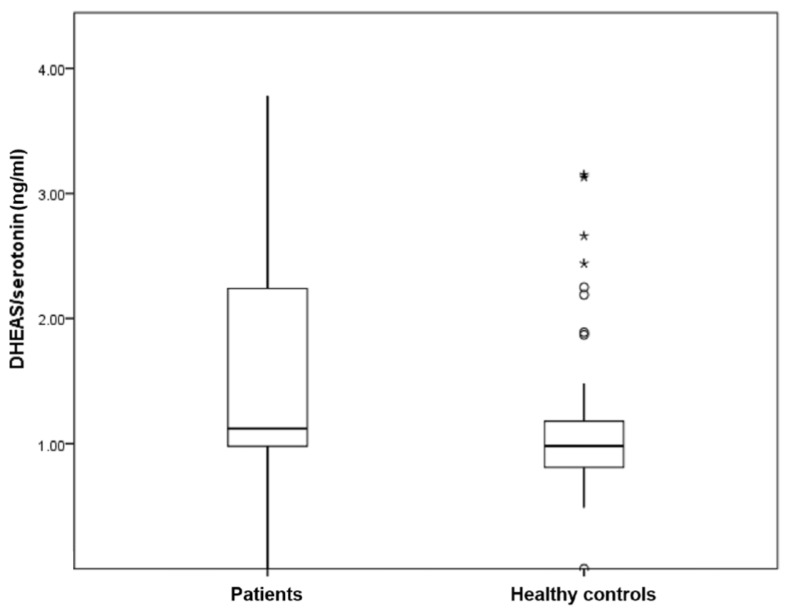
Box plots of distribution showing DHEAS/serotonin ratio in medicated patients with Major Depressive Disorder (MDD) and healthy controls.

**Figure 2:**
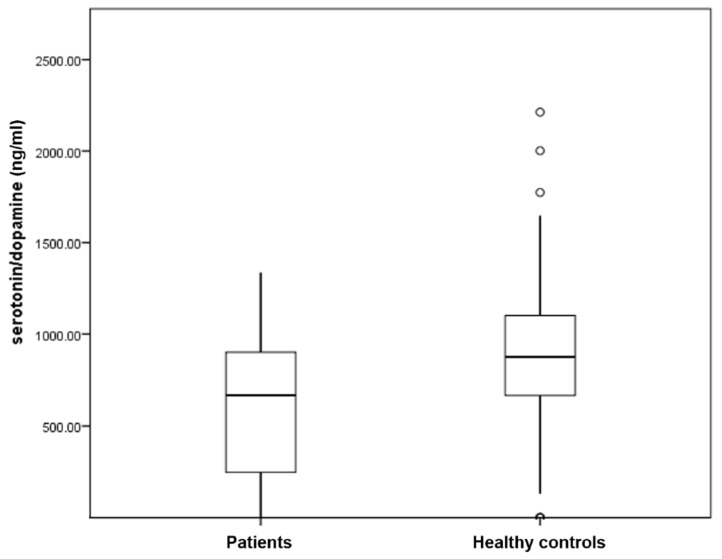
Box plots of distribution showing serotonin/dopamine ratio in medicated patients with Major Depressive Disorder (MDD) and healthy controls.

Table 2 compares the spot urine levels of monoamines and steroid hormones among medicated patients with different severities of depression, anxiety and stress. There were no significant differences in the levels of urine cortisol, serotonin and dopamine among medicated patients with different severities of anxiety, depression and stress (*p* > 0.05). The level of DHEAS was significantly higher in the moderately stress group (*p* = 0.03). Table 3 compares the spot urine levels of monoamines, steroid hormones and derived ratios between medicated patients with MDD and healthy controls. There were no significant differences in the level of urine cortisol, DHEAS, serotonin and dopamine between medicated patients with MDD and healthy controls (*p* > 0.05). Medicated patients with MDD demonstrated significantly higher spot urine DHEAS/serotonin ratio than healthy controls (1.56 vs. 1.19, *p* = 0.004) (see Figure 1). In contrast, medicated patients with MDD demonstrated significantly lower spot urine serotonin/dopamine ratio than healthy controls (599.71 vs. 888.60 *p* = 0.008) (see Figure 2).

### 3.3. Discrimination of Medicated Patients Suffering from MDD and Healthy Controls

**Figure 3:**
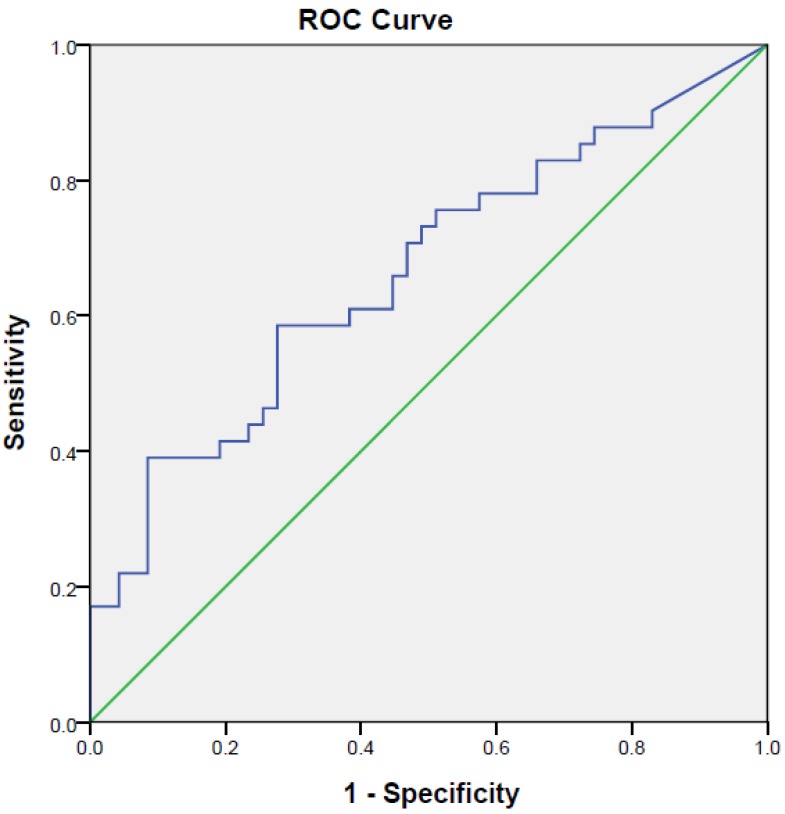
Receiver operative characteristic (ROC) curve analysis of spot urine serotonin/dopamine and differentiation of medicated patients with Major Depressive Disorder (MDD) from health controls. Spot urine serotonin/dopamine cut-off of >667.38 was the best cut-off to predict medicated patients with MDD.

**Figure 4:**
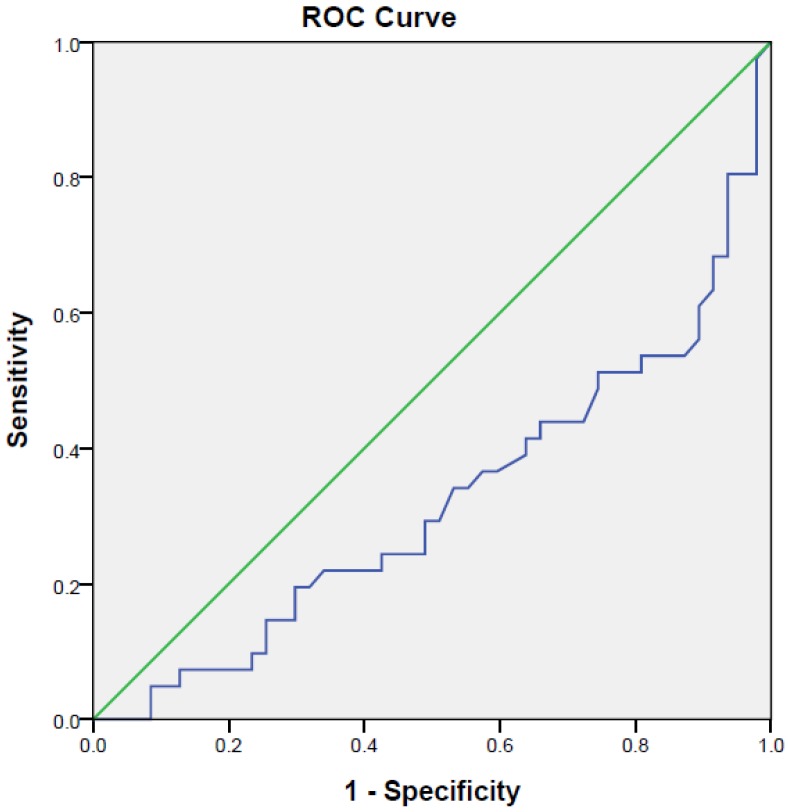
Receiver operative characteristic (ROC) curve analysis of spot urine DHEAS/serotonin and differentiation of medicated patients with Major Depressive Disorder (MDD) from health controls. The AUC for DHEA/serotonin ratio was less than 0.5. It could not discriminate medicated patients suffering from MDD and healthy controls.

Figure 3 and Figure 4 show the AUC values based on serotonin/dopamine ratio and DHEAS/serotonin ratio in distinguishing medicated patients with MDD from healthy controls. The AUC for DHEAS/serotonin ratio (AUC = 0.32) and serotonin/dopamine ratio (AUC = 0.663) demonstrated different ability to distinguish medicated patients with MDD from healthy controls. With the serotonin/dopamine cut-off of >667.38, the sensitivity was 73.2% and specificity was 51.1%. As the AUC for DHEA/serotonin ratio was less than 0.5, it could not discriminate medicated patients suffering from MDD and healthy controls.

## 4. Discussion

The main result of this study is that: (i) there were no significant differences in spot urinary levels of monoamines and steroid hormones between medicated patients suffering from MDD and healthy controls; (ii) there were no significant differences in the level of urine cortisol, serotonin and dopamine among medicated patients with different severities of anxiety, depression and stress; (iii) medicated patients suffering from MDD demonstrated significantly higher spot urinary DHEAS/serotonin ratio and lower serotonin/dopamine ratio than healthy controls; and (iv) the derived ratio of serotonin/dopamine in spot urine could differentiate medicated patients suffering from MDD when compared to healthy controls but not the DHEAS/serotonin ratio.

Our study is in accordance with a previous study showing that depressed patients did not exhibit significant change in levels of urinary monoamines as compared to healthy controls [30]. In our study, the lack of significant differences in spot urinary levels of monoamines and steroid hormones between two groups could be because medicated MDD subjects were stabilized by antidepressant treatment and the levels of urinary monoamines were not significantly lower than healthy controls. The other explanation is that most of the body’s serotonin is circulating in the peripheral bloodstream and transported by blood platelets [31]. As a result, peripheral serotonin level is less influenced by treatment of SSRIs which enhance the level of serotonin mainly in the brain. Furthermore, there were no significant differences in the levels of urine cortisol, serotonin and dopamine among medicated patients with different severities of anxiety, depression and stress. These findings suggest that there were no significant differences in the levels of urine monoamine and cortisol between responders and non-responders to antidepressants.

Ritsner et al. (2005) investigated the relationship between serum cortisol, DHEAS, cortisol/DHEAS ratio and responsiveness to antipsychotic drugs in patients suffering from schizophrenia. This study found that derived ratio (i.e., cortisol/DHEAS ratio) showed advantage over serum levels of cortisol and DHEAS for prediction of responsiveness to antipsychotic drugs [32]. They reported that serotonin/dopamine ratio could differentiate medicated patients with MDD from healthy controls. Serotonin is an inhibitory neurotransmitter which is depleted in MDD and associated with decreased immune system function [15]. Low level of serotonin is associated with anxiety, binge eating, depression, obsession and compulsion, somatic complaints and sleep disturbance. In contrast, mood disturbance occurs when dopamine is either elevated or low. In medicated subjects with MDD, the significant reduction in serotonin relative to dopamine levels suggest that antidepressant treatment could not increase the serotonin levels in synapse to counter the negative effects of fluctuating dopamine levels on mood. As a result, the derived ratio of serotonin/dopamine could be more useful than using the level of serotonin or dopamine alone to differentiate medicated patients with MDD from healthy controls. Similarly, the serotonin-to-dopamine transporter binding ratio was found to change as Parkinson disease (PKD) progresses in medicated patients suffering from PKD. The serotonin-to-dopamine transporter binding ratio was not affected by the duration of dopaminergic medication treatment.

In this study, there was no significant difference in the DHEAS/cortisol ratio between medicated patients with depression and healthy controls. In animal studies, there was no significant difference in the DHEAS/cortisol ratio between aggressive dogs and controls but the level of serotonin was found to be significantly higher in aggressive dogs [25]. These findings suggest that DHEAS/cortisol ratio was not a sensitive biomarker to detect depression and aggression. A second possible explanation is that steroid hormone is less sensitive than monoamine in detecting depression and aggression. Third, the diurnal variation of cortisol and DHEAS level could affect the result and led to non-significant finding.

There is a need in psychiatry to have an objective, non-invasive and biological approach to diagnosis prognostication and treatment selection. For example, functional near-infrared spectroscopy (fNIRS) was used to diagnose psychiatric disorders and differentiate psychiatric patients from healthy controls by measuring oxy-haemoglobin levels in cerebral blood flow [8]. The sensitivity and specificity of using integral values generated by fNIRS to differentiate medicated patients with psychiatric disorders and healthy controls were 82% and 50%, respectively [33]. In the future, the integral value of fNIRS can be combined with spot urine serotonin/dopamine ratio to enhance the accuracy to differentiate medicated patients with MDD from healthy controls. 

There were several limitations in this study: First, the number of recruited participants in this study was relatively small. Second, only a single urine sample was collected in this study. From scientific perspective, the biological variability of urine specimen collection is well recognized and 24-h urine sample collection would provide a better estimate of levels of monoamines over a period of time [27]. From clinical perspective, patients suffering from MDD would find it difficult to have 24-h urine collection and result in rejection or poor adherence. Nevertheless, previous study found that 24-h urine collection and spot urine demonstrated good correlation [27]. Third, due to the cross-sectional design of this study, we could not demonstrate causality of the observed differences between medicated patients with MDD and healthy controls. Fourth, the finding of this study is limited by ecological fallacy [34] and we could not exclude the effects of meals, exercise, sleep and smoking on urinary levels of monoamines. Finally, we did not measure creatinine level. As a result, we could not calculate creatinine-adjusted levels of monoamines and steroid hormone.

In conclusion, the application of a spot urine serotonin/dopamine ratio could be an adjunct laboratory tool to confirm the diagnosis of MDD. In the future, the methods described in this study will be applied to assess for severity of depression, effectiveness of antidepressant treatment, compliance to antidepressant treatment and prediction of the future prognosis of MDD in personalized medicine. Further studies should be performed to confirm whether spot urine serotonin/dopamine ratio could effectively separate unmedicated patients with MDD from healthy controls.

## Figures and Tables

**Table 1:** Basic characteristics of medicated patients with Major Depressive Disorder (MDD) and healthy controls.

	Medicated Patients with MDD (*n* = 47)	Healthy Controls (*n* = 41)	Statistical Test	*p*-Value
Age			*t*-test	0.104
Mean ± SD	40.65 ± 10.94	44.6 ± 11.28		
Median (min–max)	42.5 (21–61)	47 (23–62)		
Gender			Fisher’s exact	0.276
Male	15 (31.9%)	18 (43.9%)		
Female	32 (68.1%)	23 (56.1%)		
Ethnicity			Chi square	0.453
Chinese	36 (76.6%)	34 (82.9%)		
Malay	3 (6.4%)	1 (2.4%)		
Indian	6 (12.8%)	2 (4.9%)		
Eurasian	1 (2.1%)	1 (2.4%)		
Others	1 (2.1%)	3 (7.3%)		
Depression score			Mann–Whitney U	<0.001 **
Mean ± SD	19.29 ± 12.79	2.29 ± 3.24		
Median (min–max)	18 (0–42)	0 (0–12)		
Anxiety score			Mann–Whitney U	<0.001 **
Mean ± SD	18.86 ± 10.03	2.93 ± 2.69		
Median (min–max)	20 (0–42)	2 (0–8)		
Stress score			Mann–Whitney U	<0.001 **
Mean ± SD	21.26 ± 10.38	5.29 ± 4.57		
Median (min–max)	21 (0–42)	4.67 (0–18)		
Depression			Chi square	<0.001 **
Normal (0–9)	14 (29.8%)	39 (95.1%)		
Mild (10–13)	5 (10.6%)	2 (4.9%)		
Moderate (14–20)	7 (14.9%)	0		
Severe (21–27)	5 (10.6%)	0		
Extremely severe (≥28)	16 (34.0%)	0		
Anxiety			Chi square	<0.001 **
Normal (0–7)	6 (12.8%)	36 (87.8%)		
Mild (8–9)	4 (8.5%)	5 (12.2%)		
Moderate (10–14)	7 (14.9%)	0		
Severe (15–19)	6 (12.8%)	0		
Extremely severe (≥20)	24(51.1%)	0		
Stress			Chi square	<0.001
Normal (0–14)	16 (34.0%)	40 (97.6%)		
Mild (15–18)	5 (10.6%)	1 (2.4%)		
Moderate (19–25)	10 (21.3%)	0		
Severe (26–33)	9 (19.1%)	0		
Extremely severe (≥34)	7 (14.9%)	0		

* *p* < 0.05; ** *p* < 0.001.

**Table 2:** Comparison of levels of monoamines and cortisol among medicated patients with different severities in anxiety, depression and stress (*n* = 47).

	Normal	Mild	Moderate	Severe	Extremely Severe	*p*-Value
Depression						
*n*	14	5	7	5	16	
Cortisol	152.39 (50.58–409.35)	114.18 (67.91–298.55)	127.63 (0–354.45)	63.84 (50.88–224.60)	137.49 (0–331.20)	0.606
DHEAS	401.62 (90.89–764.85)	154.50 (73.09–338.3)	303.20 (117.63–599.03)	98.21 (63.67–565.93)	229.14 (82.95–735.40)	0.259
Serotonin	194.24 (70.58–495.73)	133.15 (75.58–321.35)	200.20 (0–387.98)	89.18 (66.60–229.90)	174.58 (66.83–350.25)	0.409
Dopamine	263.22 (0–494.58)	204.95 (0–447.05)	272.45 (0–478.35)	461.13 (0–1723.25)	193.00 (0–626.93)	0.422
Anxiety						
*n*	6	4	7	6	24	
Cortisol	151.52 (84.80–221.88)	138.53 (108–182.65)	162.53 (73.22–298.55)	188.90 (63.84–409.35)	104.37 (0–331.20)	0.289
DHEAS	393.63 (147.18–764.85)	410.92 (110.41–588.80)	331.58 (117.63–625.10)	318.57 (89.81–593.18)	205.90 (63.67–599.03)	0.280
Serotonin	171.63 (132.77–350.25)	186.48 (133.15–243.00)	237.40 (108.03–379.78)	200.29 (89.18–495.73)	123.23 (0–315.95)	0.205
Dopamine	278.07 (186.65–388.53)	349.165 (204.95–461.13)	242.78 (0–380.73)	486.47 (140.10–1723.25)	184.80 (0–626.93)	0.093
Stress						
*n*	16	5	10	9	7	
Cortisol	162.71 (50.58–409.35)	106.35 (63.84–354.45)	148.90 (0–298.55)	99.29 (50.88–142.15)	97.67 (0–331.20)	0.119
DHEAS	293.74 (90.89–764.85)	154.50 (73.09–333.93)	408.22 (117.63–599.03)	132.88 (63.67–590.90)	164.55 (82.95–292.18)	0.030 *
Serotonin	230.34 (70.58–495.73)	123.73 (75.58–387.98)	177.28 (0–321.35)	121.70 (66.66–200.20)	112.98 (66.83–315.95)	0.106
Dopamine	280.03 (0–626.93)	478.35 (0–1723.25)	235.13 (0–461.13)	182.90 (0–393.15)	191.75 (129.30–497.70)	0.585

Data were reported as median (min–max). Kruskal–Wallis analysis was performed unless specified otherwise. * Mann–Whitney U test was done to compare DHEAS levels between stress severity categories in a pairwise manner. *p* ≤ 0.05 for the following pairs: normal and severe, normal and extremely severe, moderate and severe, moderate and extremely severe.

**Table 3:** Comparison of monoamines, steroid hormones and derived ratios between medicated patients with MDD and healthy controls.

	Medicated Patients with MDD (*n* = 47)	Healthy Controls (*n* = 41)	Statistical Test	*p*-Value
Cortisol (ng/mL)			Mann–Whitney U	0.533
Mean ± SD	148.56 ± 87.50	172.47 ± 117.90		
Median (min–max)	142.15 (0–409.35)	128.45 (0–604.50)		
DHEAS (ng/mL)			Mann–Whitney U	0.607
Mean ± SD	298.32 ± 203.63	247.05 ± 149.86		
Median (min–max)	244.00 (63.67–764.85)	229.00 (0–757.63)		
Serotonin (ng/mL)			Mann–Whitney U	0.281
Mean ± SD	187.10 ± 102.48	236.83 ± 210.55		
Median (min–max)	170.68 (0–495.73)	168.55 (0–1315.25)		
Dopamine (pg/mL)			Mann–Whitney U	0.682
Mean ± SD	277.70 ± 271.97	280.19 ± 251.81		
Median (min–max)	242.98 (0–1723.25)	216.10 (0–1348.83)		
Cortisol/DHEAS			Mann–Whitney U	0.252
Mean ± SD	0.603 ± 0.310	0.700 ± 0.282		
Median (min–max)	0.670 (0–1.18)	0.620 (0–1.32)		
Cortisol/Serotonin			Mann–Whitney U	0.116
Mean ± SD	0.771 ± 0.200	0.876 ± 0.639		
Median (min–max)	0.7800 (0–1.10)	0.610 (0–2.25)		
Cortisol/Dopamine			Mann–Whitney U	0.171
Mean ± SD	463.95 ± 315.04	729.86 ± 646.77		
Median (min–max)	492.63 (0–1084.22)	575.01 (0–3126.00)		
DHEAS/Serotonin			Mann–Whitney U	0.004 *
Mean ± SD	1.56 ± 0.864	1.19 ± 0.702		
Median (min–max)	1.12 (0–3.78)	0.980 (0–3.15)		
DHEAS/Dopamine			Mann–Whitney U	0.363
Mean ± SD	972.71 ± 903.73	1047.86 ± 806.38		
Median (min–max)	709.99 (0–4057.54)	885.03 (0–3986.73)		
Serotonin/Dopamine			Mann–Whitney U	0.008 *
Mean ± SD	599.71 ± 394.56	888.60 ± 528.75		
Median (min–max)	665.39 (0–1335.20)	875.89 (0–2212.59)		

* *p* < 0.05.

## Supplementary Materials

The following are available online at http://www.mdpi.com/1660-4601/15/5/865/s1.
