# Supplementary material for: Differentiating Medicated Patients Suffering from Major Depressive Disorder from Healthy Controls by Spot Urine Measurement of Monoamines and Steroid Hormones
Source: Int J Environ Res Public Health. 2018 Apr 26;15(5):865. doi: 10.3390/ijerph15050865 (PMC5981904; doi:10.3390/ijerph15050865)
Supplement: Supplementary file 1 [file ijerph-15-00865-s001.pdf]

Table S1. Information about antidepressant treatment (n = 47).

Name of antidepressant	Number of patients (%)	Mean dose + Standard Deviation)
**Selective Serotonin Reuptake Inhibitors (SSRIs)**		
Fluoxetine	7 (14.9%)	35.71 ± 22.99 mg/day
Fluvoxamine	12 (25.5%)	106.25 ± 69.19 mg/day
Escitalopram	5 (10.6 %)	16 ± 5.48 mg/day
Paroxetine	4 (8.5%)	25 ± 17.6 mg/day
Sertraline	4 (8.5%)	56.25 ± 31.46 mg/day
**Serotonin Noradrenaline Reuptake Inhibitor (SNRI)**		
Venlafaxine	2 (4.3%)	187.5 ± 53.03 mg/day
**Noradrenergic and Specific Serotonergic Antidepressant (NaSSA)**		
Mirtazapine	17 (29.8%)	18.97 ± 11.29 mg/day
**Dopamine Noradrenaline Reuptake Inhibitor (DNRI)**		
Bupropion	3 (6.4%)	150 ± 0 mg/day
**Other antidepressants**		
Agomelatine	3 (6.4%)	41.67 ± 14.43 mg/day
Vortioxetine	1 (2.1%)	10 ± 0 mg/day
**Combination of antidepressants (n = 13)**		
Agomelatine + Fluoxetine (n = 1)<br>Agomelatine + Fluvoxamine (n = 1)<br>Agomelatine + Mirtazapine (n = 1)<br>Bupropion + Mirtazapine (n = 2)<br>Bupropion + Fluvoxamine (n = 1)	Escitalopram + Venlafaxine (n = 2)<br>Mirtazapine + Paroxetine (n = 2)<br>Mirtazapine + Fluvoxamine (n = 1)<br>Mirtazapine + Vortioxetine (n = 1)<br>Paroxetine + Sertraline (n = 1)	
